# Supplementary material for: A Compared Study of Eicosapentaenoic Acid and Docosahexaenoic Acid in Improving Seizure-Induced Cognitive Deficiency in a Pentylenetetrazol-Kindling Young Mice Model
Source: Mar Drugs. 2023 Aug 24;21(9):464. doi: 10.3390/md21090464 (PMC10533149; doi:10.3390/md21090464)
Supplement: Supplementary file 1 [file marinedrugs-21-00464-s001.zip › marinedrugs-2521134-supplementary.pdf]

Table S1. Ingredient and main fatty acid compositions of experimental diets

**Ingredient(g/kg)**	**AIN93G**	**EPA**	**DHA**
Corn starch	397.5	397.5	397.5
Dextrin	132	132	132
Sucrose	100	100	100
Casein	200	200	200
Powdered cellulose	50	50	50
Soybean oil	70	60	60
Mineral mix	35	35	35
Vitamin mix	10	10	10
Choline bitartrate	2.5	2.5	2.5
L-Cystine	3	3	3
EPA ethyl ester	-	10	-
DHA ethyl ester	-	-	10
t- buthlhydroquinone	0.014	0.014	0.014
Fatty acids composition (%)			
C16 :0	11.76	9.6	10.18
C18 :0	4.06	3.3	3.61
C18 :1	23.9	19.4	20.3
C18 :2n-6	48.68	38.0	40.25
C18 :3n-3	5 72	4 5	4 79
C20 :4n-6	-	1.1	-
C20 :5	-	17.0	-
C22 :6	-	2.2	17.31

Note :"-" , none detected. aModified AIN-93G.

Table S2 Parameters of neurotransmitter for MS condition.

Name	Abbreviation	Ion mode	precursor ion (m/z)	product ion (m/z)	NCE (V)

Glutamate	Glu	+	148	130	40
γ-aminobutyric acid	GABA	+	104.1	87	40
